# Neuronal Chains for Actions in the Parietal Lobe: A Computational Model

**DOI:** 10.1371/journal.pone.0027652

**Published:** 2011-11-28

**Authors:** Fabian Chersi, Pier Francesco Ferrari, Leonardo Fogassi

**Affiliations:** 1 Institute of Science and Technology of Cognition, CNR Rome, Rome, Italy; 2 Department of Neuroscience, University of Parma, Parma, Italy; 3 Department of Evolutionary and Functional Biology, University of Parma, Parma, Italy; 4 Department of Psychology, University of Parma, Parma, Italy; 5 Italian Institute of Technology (RTM), Parma, Italy; Duke University, United States of America

## Abstract

The inferior part of the parietal lobe (IPL) is known to play a very important role in sensorimotor integration. Neurons in this region code goal-related motor acts performed with the mouth, with the hand and with the arm. It has been demonstrated that most IPL motor neurons coding a specific motor act (e.g., grasping) show markedly different activation patterns according to the final goal of the action sequence in which the act is embedded (grasping for eating or grasping for placing). Some of these neurons (parietal mirror neurons) show a similar selectivity also during the observation of the same action sequences when executed by others. Thus, it appears that the neuronal response occurring during the execution and the observation of a specific grasping act codes not only the executed motor act, but also the agent's final goal (intention).

In this work we present a biologically inspired neural network architecture that models mechanisms of motor sequences execution and recognition. In this network, pools composed of motor and mirror neurons that encode motor acts of a sequence are arranged in form of action goal-specific neuronal chains. The execution and the recognition of actions is achieved through the propagation of activity bursts along specific chains modulated by visual and somatosensory inputs.

The implemented spiking neuron network is able to reproduce the results found in neurophysiological recordings of parietal neurons during task performance and provides a biologically plausible implementation of the action selection and recognition process.

Finally, the present paper proposes a mechanism for the formation of new neural chains by linking together in a sequential manner neurons that represent subsequent motor acts, thus producing goal-directed sequences.

## Introduction

The inferior parietal cortex has been traditionally conceived as a typical association cortex [1], [2], because of its polymodal neuronal properties and of the occurrence of spatial deficits after its damage in humans. However, neurophysiological [3]–[5] and lesion [6]–[8] studies have demonstrated that this cortical sector is also involved in motor control. In particular, neurons of the inferior parietal lobule (IPL) are active during execution of several types of goal-related motor acts, such as reaching, grasping, bringing to the mouth or during eye movements and many of them integrate sensory and motor properties [3], [5], [9]. This integration subserves several types of sensorimotor transformations for reaching, grasping, oculomotion [10]–[12].

An example of parietal neurons integrating sensory and motor properties is represented by mirror neurons [5], [13]–[15] that, similarly to those previously found in ventral premotor cortex (PMv), are active during execution of goal-related motor acts and during observation of similar motor acts performed by another individual. It has been proposed that this matching mechanism underpins understanding of the observed motor acts.

**Figure 1:**
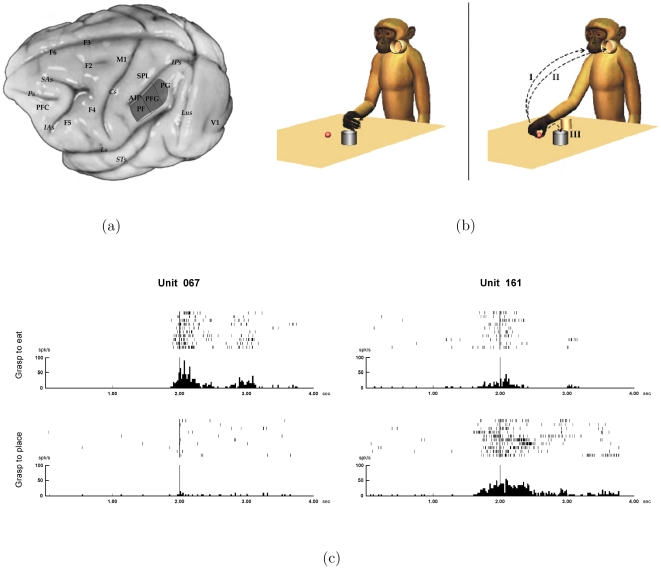
Details of the experiment of Fogassi et al. [**14**]. (a) Lateral view of the monkey brain showing the sector of IPL (dark shading) from which the neurons were recorded. (b) The apparatus and the paradigm used for the motor task. I. grasping for eating. II. and III. grasping for placing inside a container put near the mouth or near the target, respectively. (c) Activity of two IPL neurons during the two grasping conditions. Rasters and histograms are aligned with the moment when the monkey touched the object to be grasped. x axis, time, bin 20 ms; y axis, discharge frequency (spikes per second).

An interesting issue is whether neurons of IPL could have a role in coding not only motor acts but also actions. Here action is defined as a sequence of motor acts aimed at a specific final goal. In order to address this question, a study [14] investigated the activity of IPL neurons (see Figure 1a) in monkeys trained to perform a motor task, consisting of two main conditions. In one condition, the monkey, starting from a fixed position (Figure 1b), reached for and grasped a piece of food located on a table and brought it to the mouth. In another condition, the monkey reached for and grasped an object located on the table and then placed it into a container. Note that the initial reaching-grasping act is common to both conditions.

Grasping neurons of IPL were recorded while the monkey performed both conditions of the task. The results showed that during grasping execution the discharge of the majority of these motor neurons was modulated by the final goal of the action. Some neurons discharged stronger during grasping for eating, others during grasping for placing (Figure 1c). The remaining neurons did not show any modulation.

In order to explain the peculiar behavior of these grasping neurons it has been hypothesized that their differential discharge reflects the motor intention of the agent. Furthermore, since it is known that every motor act belonging to an action is fluently coordinated with the preceding and the subsequent one [16], [17], it has been proposed [14], [18], [19] that in IPL actions are coded by neuronal chains, each of which leading to a specific action goal, that corresponds to the motor intention of the acting agent. In every chain, each neuron coding a motor act facilitates the activation of the neuron coding the next motor act of the chain, thus providing motor fluency to the whole action. It has been hypothesized that in this type of action organization it is necessary to postulate the presence of a “selection mechanism” that chooses the dedicated neuronal action chains, based on information provided by a given context and by previous experience. In fact, the selection of a specific chain depends on several contextual factors such as the type of target object, the setting in which the action takes place and internal motivational factors [15]. It has been proposed that this selection mechanism is localized in the ventral prefrontal cortex, because of its role in integrating and processing these factors [20] and of its connections with IPL [21]–[23]. These connections could constitute the anatomical pathway through which prefrontal cortex, on the basis of contextual information and previous experience, could recruit specific parietal chains.

In the study of Fogassi et al. [14] it was also investigated whether the discharge of IPL mirror neurons can be modulated by the final goal of an observed action. The same task used for studying the behavior of IPL motor neurons was performed by an experimenter in front of the observing monkey. The experimental conditions were the same as those of the motor task: the experimenter grasped a piece of food for eating it and grasped an object for placing it.

**Figure 2:**
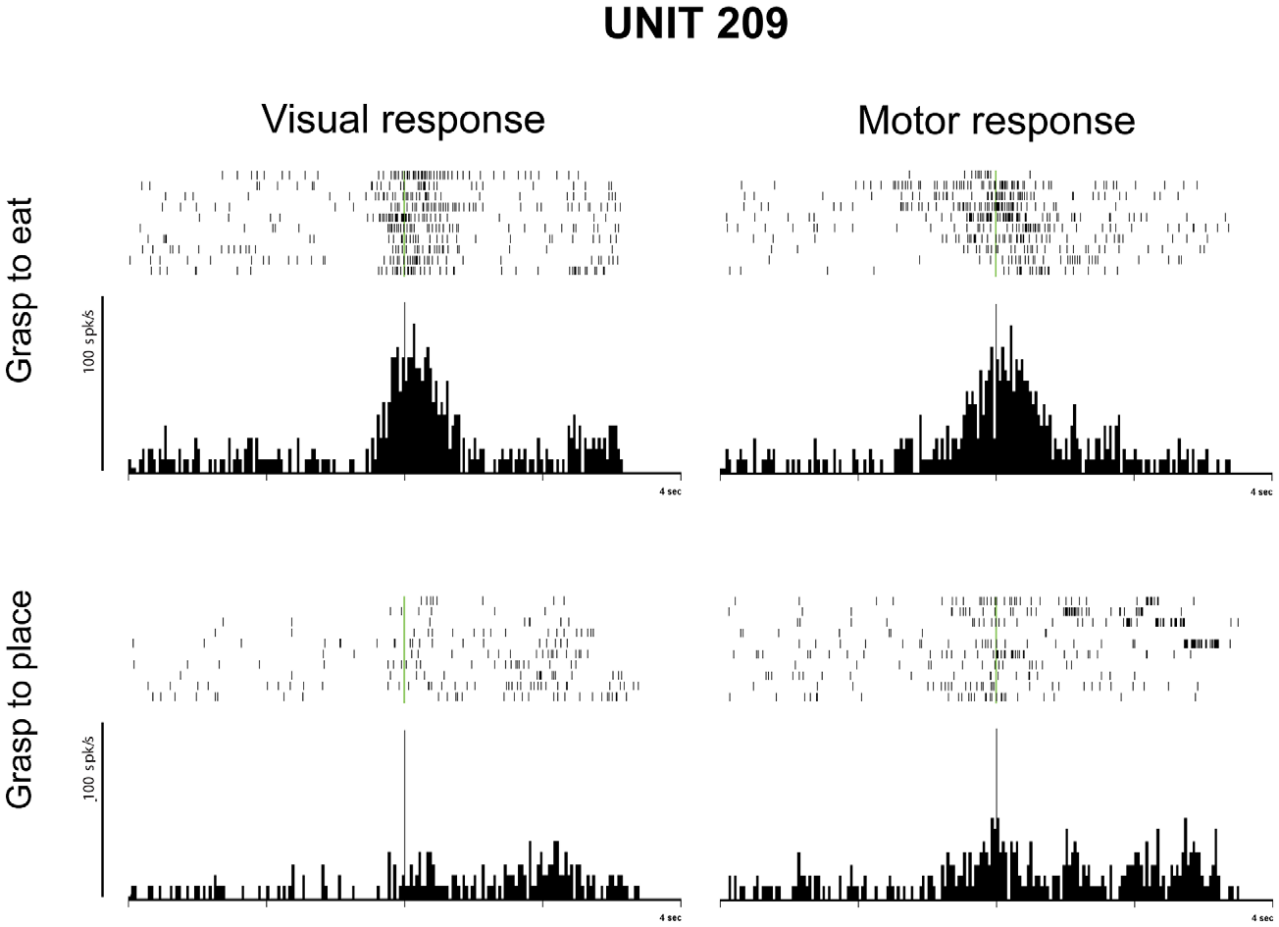
Differential discharge of a mirror neuron during the motor and the visual task. Rasters and histograms are synchronized with the moment when the monkey or the experimenter touched the object to be grasped. Other conventions as in figure 1.

The results showed that, similarly to motor neurons, the great majority of mirror neurons was differentially activated during the visual task depending on whether the observed grasping was followed by bringing to the mouth or by placing (Figure 2, left). Since the visual discharge occurs during observation of grasping, hence before the beginning of the next motor act (bringing to the mouth or placing), the differential visual discharge of mirror neurons seems to predict the final goal of the action. Furthermore, comparing the neuron response during the visual and the motor task, it was clear that the differential modulation during observation of grasping in the two different conditions was congruent with that recorded during action execution (Figure 2, right). According to the chain model, the observation of a motor act activates in the observer the motor representation of that motor act, which is embedded in a specific neuronal action chain. The retrieval of this motor act representation, that is linked to a specific action goal, would therefore allow the observer to predict the intention of the observed agent.

Neurophysiological findings [5] revealed in IPL a somatotopy of motor acts along its rostro-caudal axis, with the mouth represented more rostrally, followed caudally by the hand, the arm, and the eyes fields. The finding that these cortical fields are anatomically connected [23] gives a support to the proposed chained structure underlying action organization. The aim of this study is to provide a detailed mathematical model of the hypothesized chained organization of IPL. Simulations show that the model is able to accurately reproduce the neurophysiological data of IPL motor and mirror neurons.

## Results

### The Cortical Network Model

**Figure 3:**
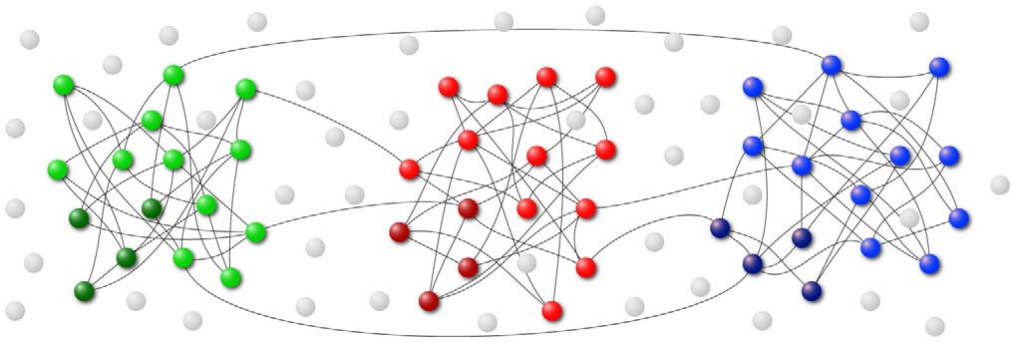
Schematic representation of three connected neuronal pools. Inhibitory neurons are represented as darker elements while excitatory neurons are represented as lighter elements.

We modeled the investigated IPL region as a two-dimensional layer of spiking neurons, grouped into small local pools (500 units) that are strongly interconnected, share the same properties (i.e. they code the same motor act and receive the same sensory inputs) and fire in a coherent way. Furthermore, these neurons possess also few long-range connections reaching other pools, in a configuration known as “small world” network [24] (see Figure 3). This type of configuration is considered to match the connectivity in real neural systems better than either local or random connectivity, and to optimize the ratio between neurons and connections.

The structure of the network employed in this study was motivated by anatomo-functional evidence suggesting the organization of neural circuits into assemblies of cortical neurons, that possess strong excitatory and inhibitory interconnectivity both locally and, less strongly, between different cortical areas [25], [26]. A characteristic property of this arrangement is that neurons tend to form local assemblies (pools) that respond in a similar way to incoming stimuli. This behavior could match the anatomo-functional organization of the inferior parietal cortex, as described in various studies [5], [23], [27], [28].

In our implementation, 25% of the neurons are randomly chosen to be inhibitory. Each neuron is connected to 20% of the neurons in its pool, and excitatory neurons are also connected to neurons belonging to other pools.

At initialization, input and output connections coming from or going to sensory and motor areas are randomly assigned to each pool of the IPL layer. For sake of simplicity we assumed that each neuron in the network has a specific motor selectivity, i.e. it codes only one specific motor act (i.e. reaching, grasping, etc.), which is in good agreement with available neurophysiological data [5], [14].

External inputs, such as visual, proprioceptive and other feedback stimuli, are simulated by means of incoming trains of spikes directed to specific pools.

**Figure 4:**
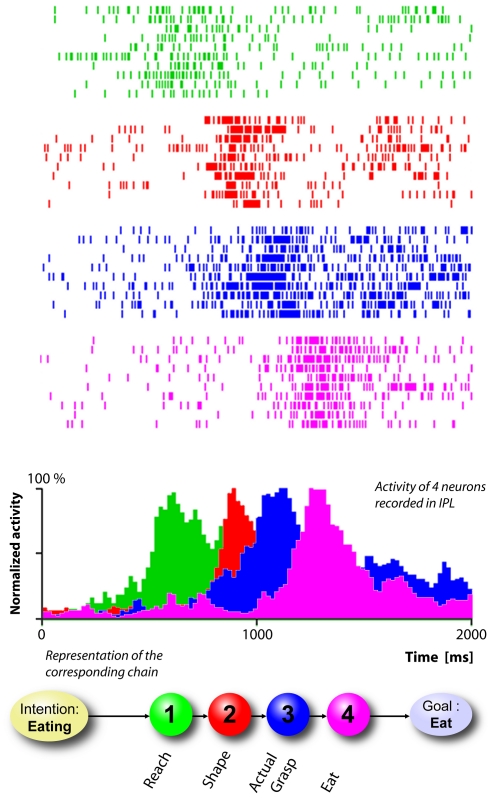
Time course of the activity (rasters and histograms) of 4 neurons recorded in IPL. Each one codes a specific motor act, but is active only when the monkey executes the “grasping to eat” sequence. Both rasters and histograms are aligned with the moment in which the monkey touches the object. Beneath the histograms a schematic representation of the corresponding neuronal chain is shown.

The core concept of the present model is that the pools coding subsequent motor acts of an action are connected in chains leading to the achievement of a specific action goal (Figure 4). In this view, although two action sequences (grasp-to-eat and grasp-to-place) may have in common several motor acts (reaching, shaping, grasping), the two corresponding neuronal chains contain physically distinct pools coding the same motor acts but each chains is specific for a particular goal.

### Mechanism of activity propagation in the chain

**Figure 5:**
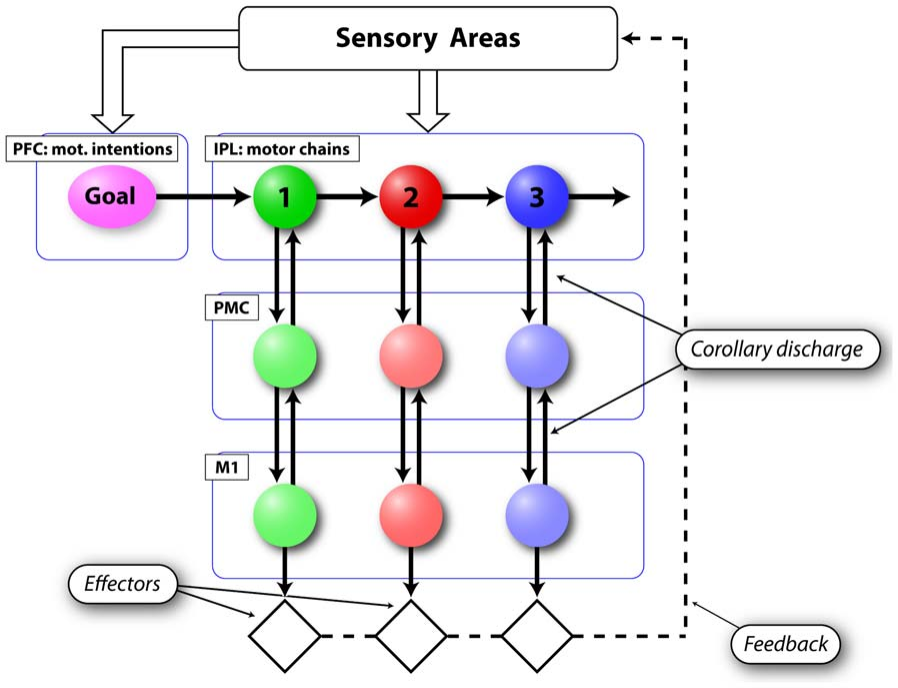
Connection scheme for one neuronal chain. Each colored circle represents a pool of neurons that codes a specific motor act. Pre-frontal input triggers the activation of the chain, while sensory and motor signals modulate the propagation of the activity within the chain. This scheme shows the path of the motor commands (from IPL to PMC to M1) and of the efferent copies (from M1 to PM to IPL). Sensory information (dashed line), which results from the interaction of the individual with the environment, follows an indirect route through sensory areas. M1 = primary motor cortex; PMC = premotor cortex; PFC = prefrontal cortex.

Figure 5 shows the scheme for a single IPL chain and its connections with other areas. Let's consider one of the sub-populations of neurons composing an IPL chain, for example pool number 2. It receives an input from the previous pool of the chain, i.e. pool number 1, and transmits its output to the following neurons (pool number 3). Additionally, it sends its output to the pre-motor areas (PMC) from which at the same time it receives a motor corollary discharge. Sensory inputs (visual contextual information, hand visual feedback, somatosensory feedback) and motor signals are necessary to synchronize the propagation of activity waves within the chains with the constraints of the physical world.

This organization allows the “smooth” and automatic execution of motor sequences because spiking activity related to single motor acts can directly propagate from one sub-population of the chain to the next in a synchronous wave of neuronal firing.

The overt motor output results from the transmission of the activity from IPL to pre-motor and primary motor cortex. The function of PMC is that of retrieving the appropriate motor acts from its internal motor vocabulary [29] while the role of primary motor cortex is that of implementing the single movements composing each motor act.

In more detail, we can formalize the total input to a generic neuron in the chain as:
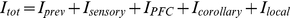
(1)where 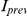 is the input from the previous neuron. If the neuron is the first of the chain then this contribution is equal to zero because it receives input only from neurons in PFC. 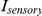 is the input from sensory areas which comprises visual, auditory and somatosensory signals. 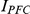 is the signal coming from prefrontal cortex (PFC). For sake of simplicity, in the proposed model this contribution is absent for neurons that are not the first ones in the chain. However, we cannot exclude that PFC sends signals also to other pools of the sequence during action unfolding. As will be explained later in detail, in our model we attribute to PFC the role of evaluating and integrating environmental cues, past events and motivation, in order to produce the activation of specific chains. In other words, the module labeled “PFC” is responsible of the selection of neuronal chains through a mechanism which here is referred to as the formation of “motor intention”.


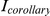
 is the signal coming back from premotor and motor areas and conveying information about the ongoing action. This signal is needed during motor execution because it confirms that the imparted command is being correctly executed and triggers the transition of the activity pulse. The rich anatomical reciprocal connections between IPL and PMC [12], [15], [23] is most likely the route through which signals are conveyed.


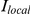
 is the total input deriving from locally connected neurons. In our simulation this input is not strong enough to create auto-sustained activity, but it stabilizes and enhances local signals.

The behavior of neuronal pools is highly non-linear both because of the complex internal dynamics of the neurons (themselves non-linear in nature) and because of their mutual interactions. The 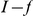 response function has a typical sigmoidal shape. The transition between low and high firing rates is due to the sudden activation of majority of the neurons in a pool (avalanche effect). This threshold depends on parameters of the network such as connectivity strength, ratio between excitatory and inhibitory neurons, and neuronal time constants.

In the following sections we will describe the circuit that is active during the motor and the visual task, and report the results of the corresponding simulations.

### Motor Task

**Figure 6:**
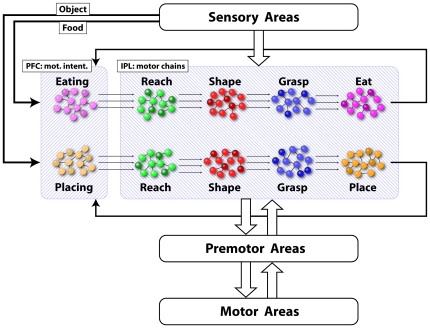
Schematic representation of the areas and the populations of neurons that are active during the motor task. The prefrontal cortex contains the motor intentions (in the figure: mot. intent.) and thus acts as the chain selector, while sensory and motor corollary signals regulate the transmission of activity waves within the chains.

The motor task used for the simulation of the model proposed in the present study consists in the execution of a “reaching-grasping-eating” or a “reaching-grasping-placing” motor sequence depending on whether the target to be grasped is a piece of food or a non edible object. Figure 6 shows a schematic representation of the network that is involved during the motor task.

The scheme is composed of several modules. The main structure is represented by the two chains of neurons contained in the large shaded rectangle that represents the IPL region. Neurons in these chains are either purely motor or visuomotor (mirror). Indeed mirror neurons contribute only with their motor response. Neurons of the chains receive auditory, visual and somatosensory inputs from sensory areas that convey information about the environment and the ongoing events. For example they receive visual information about the position and the type of target, the position of the acting hand in relation to the target and somatosensory information about the contact of the hand with the target. These and other inputs regulate and synchronize the transmission of activity patterns within the chains.

The selection of a specific action goal is expressed by the high activity level of a specific neuronal pool in PFC, here referred to as “intention pool”. The output of this pool then reaches the first element of the connected chain (see below) in form of a high frequency burst of activity.

In case of ambiguity when the cues in the scene do not permit to clearly establish the final goal of the task because they are either not sufficiently informative or too many and conflicting (for example when the object is not visible because hidden inside a container), all the intentions compatible with the given conditions are simultaneously activated and multiple chains are started in parallel. This appears to have a neurophysiological validity, since it has been reported that when the monkey at the beginning of the action has no sufficient cues to decide which action (eating or placing) to perform, during the first grasping act the differential response disappears [30]. The simultaneous activation of both chains does not represent a problem for action execution. In fact, even if a certain degree of uncertainty can be present at the moment in which the chains are activated, very likely during action unfolding additional information will become available to the agent, and the activity in the mismatching chain will die out, while neuronal activity will continue to be transmitted through the chain that is compatible with the external information, thus producing the motor output appropriate for the correct goal achievement.

The transmission of activity along the chains during the motor task occurs as follows (see Figure 6): When the intention pool is activated in PFC and the “go signal” (consisting in the lifting of a barrier placed between the target and the monkey) is given, a wave front of activity is transmitted from PFC to the first pool of the selected chain (i.e. the reaching pool). In the moment in which neurons of the first pool begin to fire, this activity is transmitted to the connected premotor and primary motor cortices, which in turn start the reaching movement. Corollary discharge signals, following the inverse path, reach the parietal areas, and contribute to sustain the activity of neuronal pools as long as the motor act is being executed. At the same time, spikes are transmitted to the neurons of the subsequent pool (i.e. the shaping pool). This input alone is not sufficient to bring the pool from its default resting state to the excited state but brings it only to a subthreshold level. The pool will reach the threshold activity level only when it receives additional sensory inputs signaling that the hand is in proximity of the object. The need of additional inputs from other external sources ensures that activity does not propagate instantaneously or in an uncontrolled manner. Neurophysiological data show that the activity of the neural pools coding different motor acts is smoothly overlapping in time (see Figure 4). It is likely that the repeated firing of the neurons of one pool contributes to the gradual build-up of activity in the following pool. Although in our model neurons possess a specific internal dynamic, time is not explicitly coded within the chains structure or the neuronal connections. The rising and falling of activity of a population is only partially determined by internal dynamics and mainly modulated by sensory inputs that are external to IPL. Nevertheless, the configuration of the neuronal connections (that we assume to be unidirectional) produces a temporal hierarchy among connected pools of the chain, i.e. there is a preferred direction of propagation of activity.

The successful completion of the task is the result of the activation of the correct sequence of motor acts populations which in turn corresponds to the transmission of the activity wave from the beginning to the end of the selected chain. The final output of the successful chain (accomplishment of the last motor act) is then utilized by the PFC to learn the association rules between cues and motor sequences.

### Simulation 1

In this first simulation we show how activity bursts propagate within a simple chain-structured network. The chosen experimental paradigm is the “grasping to eat” motor task.

Since in this case we were not studying learning mechanisms, the network connection weights were set in such a way to obtain a chain composed by the sequential connection of four neuronal pools corresponding to the “reaching”, “shaping”, “grasping” and “bringing to the mouth” motor acts. In our setup using an automatic fitting procedure we set 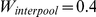.

Information about the presence of a piece of food was simulated as a train of impulses from a virtual visual module to the PFC module (Figure 6 upper left connections) lasting 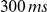.

**Figure 7:**
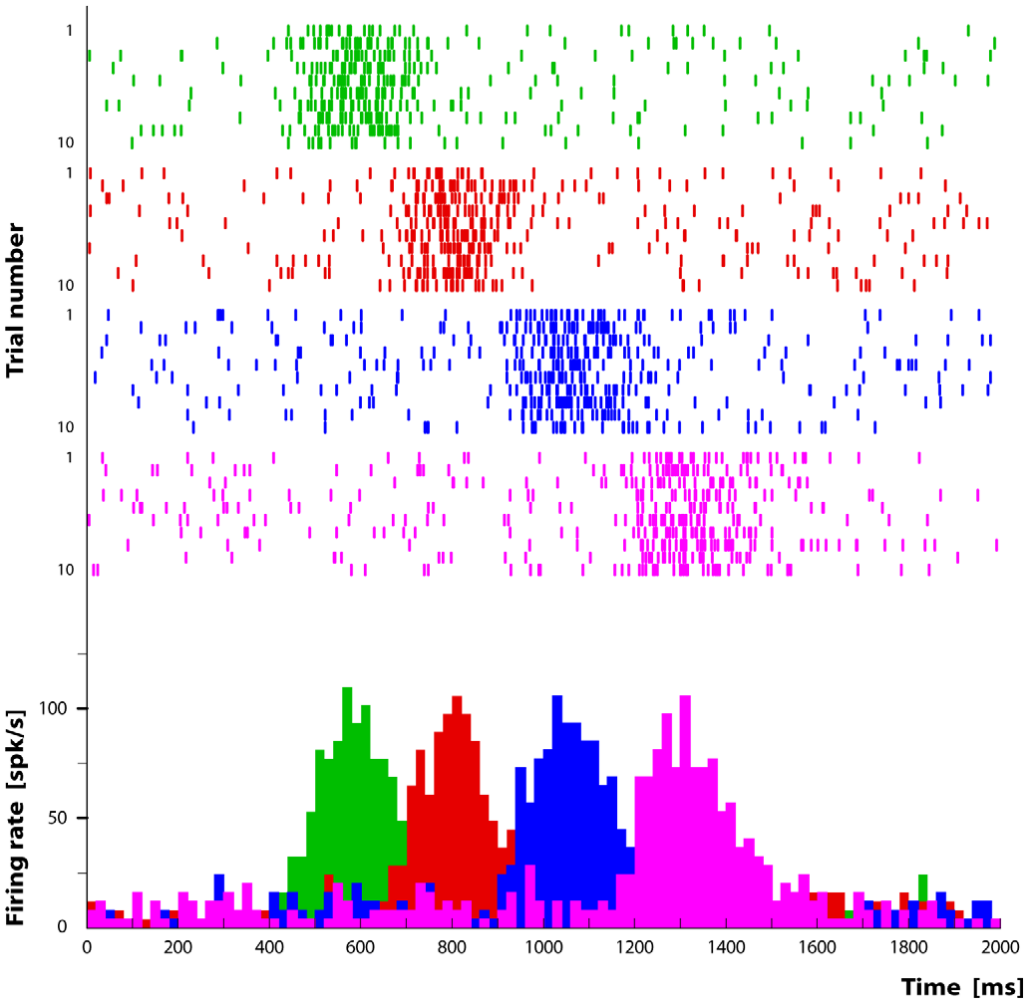
Representation of the time course of the activity patterns of four neurons of the simulated chain. The colored histograms represent the activity of neurons coding different motor acts: the green peak represents “reaching”, the red “shaping”, the blue “grasping” and the magenta “bringing to the mouth”. Both rasters and histograms are aligned with the moment (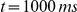) in which the simulated monkey touches the object.

The signal from the PFC module, indicating the intention to execute the “grasping to eat” action, consisted in a bell-shaped 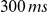 spike train with 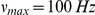 reaching 20% of the neurons of the “reaching” population in the IPL module. Due to the local connectivity this signal is rapidly transmitted to the remaining neurons in the pool giving origin to an avalanche response of the local population. The characteristic bell-shaped activity profile is due to the fact that after the external input ceases, local activity reverberates with the single pools but eventually dies out. Additionally, a fraction of the activity propagates from one pools the connected ones producing the activation of new pools (see Figure 7).

Motor execution was simulated by providing proprioceptive feedback and corollary discharge signals in form of trains of impulses of the duration of 300 ms. This value has been chosen in accord with the duration of real reaching, grasping and bringing-to-the-mouth movements (see Figure 4).

Figure 7 shows the sequential activation of the above mentioned four neuronal pools. It is clear from this figure that it is possible to build a network that can transmit coherent activity patterns along the chain. Moreover, since the propagation is regulated by external events, it is possible to stretch or compress the duration of the signal propagation without modifying the network configuration, so that the simulation speed can be adapted to actions of different duration.

### Simulation 2

**Figure 8:**
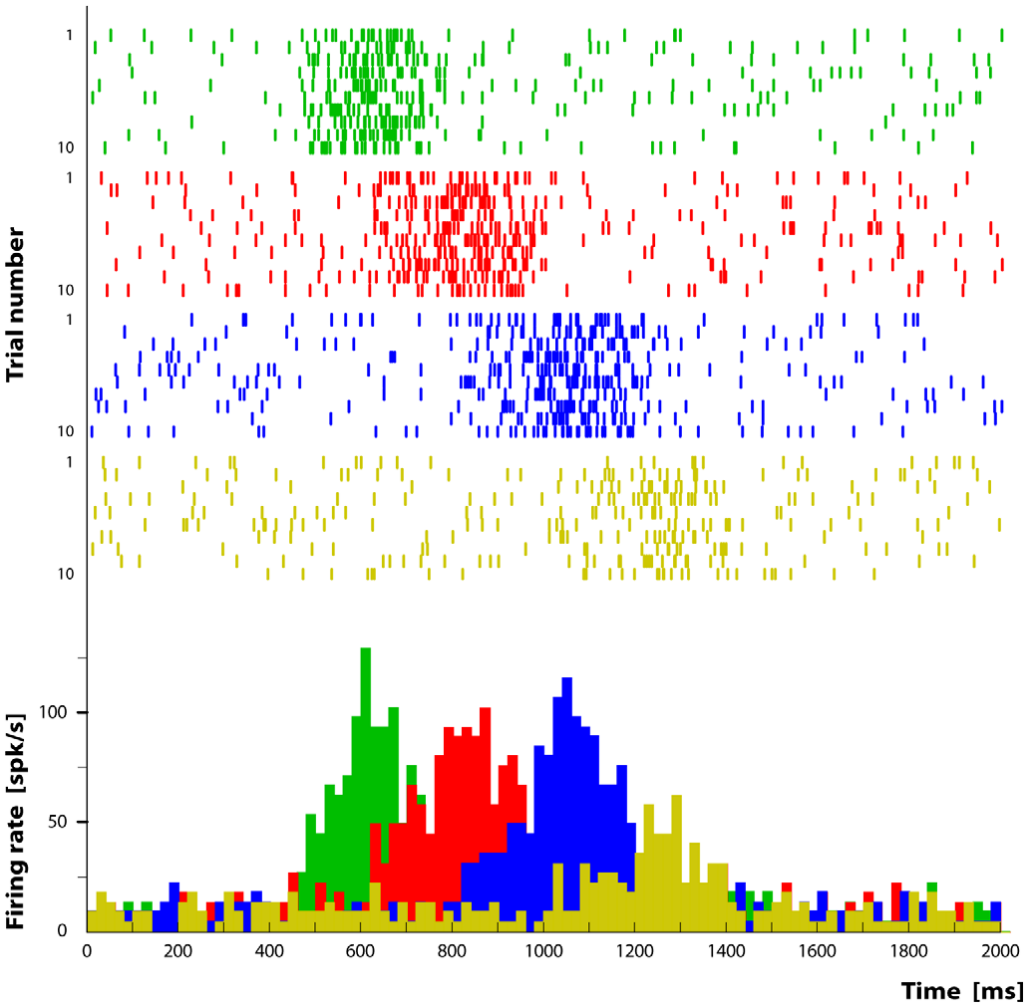
Representation of the activity of the pools forming the “reaching to place” chain during a “reaching to eat” task. The color indicates the neuronal activity related to the type of motor act (green = “reaching”, red = “shaping”, blue = “grasping”, orange = “placing”). Both rasters and histograms are aligned with the moment (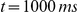) in which the simulated monkey touches the object.

In the present simulation we aimed at creating a situation of ambiguity in which the information about the target object is not available since the beginning of the task (blind task). In this task the monkey has to grasp an object hidden inside a container on the table without knowing which is the object to be grasped. It can be either a piece of food or a metal cube. After grasping and having touched the target object, the monkey receives the necessary cues to perform the remaining part of the action sequence. Since at the beginning of the task it is unknown to the agent which one of the two objects is located inside the container, the network is built in such a way that the “Intention” module activates both possible chains, i.e. the one corresponding to eating and the one corresponding to placing. When the system is started, first the “Reaching” pool, then the “Shaping” and the “Grasping” pools become active in parallel in both chains. This happens because the final action goal cannot be determined until the monkey has grasped and, therefore, recognized the object. In this specific setup, the result of the grasping act is that the object is recognized, either by touching or by seeing it, and consequently the goal of the trial is disambiguated. At this point only one of the chains represents the correct goal (in the specific example “eating”) and the other will be interrupted (see Figure 8). In our model this is automatically achieved because, once the object is identified, the sensory input will reach only one element of the chain (in this example the visual input “piece of food” reaches the “bringing to the mouth” element), thus causing the activity to propagate along one chain and die out in the other one.

### Visual Task

In the second part of the study by Fogassi et al. [14] the monkey had to observe the demonstrator either grasping a piece of food for eating it or grasping an object for placing it.

According to our hypothesis the chains involved in this task are the ones composed by neurons endowed with visual properties, i.e. mirror neurons. Note that the peculiar characteristic of mirror neurons allows their recruitment both during the motor and the visual task.

**Figure 9:**
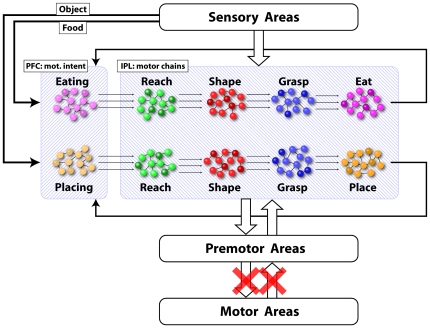
Schematic representation of the areas and the population of neurons that are active during the visual task.

In comparison to the motor task (Figure 6) the scheme for the visual task (Figure 9) contains two important differences. The first is that in this task the observer (the monkey) can only make predictions about what the demonstrator is going to do based on the cues present in the working environment. The presence of food on the table leads the monkey to predict that, with high probability, the observed agent will execute a grasping for eating action, while the presence of an object will very likely lead to a grasping for placing action.

The second difference is that the output of the pools directed to the pre-motor areas is not transmitted to the primary motor areas. An inhibitory mechanism blocks the propagation of motor commands after pre-motor areas and no overt motor output is produced. The existence of a suppressing neurophysiological mechanism has been indirectly evidenced by the lack of EMG activity during action observation [31], [32] and by the presence of mirror neurons that inhibit their discharge during observed grasping [33].

The chain activation mechanism occurring during action observation is the same as that described for the motor task. The chains composed by mirror neurons used in the Motor Task to produce motor sequences, are used in the Visual Task to recognize actions performed by other individuals by mapping the visual input onto prewired motor patterns of mirror neurons. The visual input causes the firing of mirror neurons for a specific motor act and their activity immediately propagates to population of mirror neurons coding the subsequent motor acts thus triggering an avalanche response. The proposed recognition mechanism, indicated as “hypothesis validation”, works as follows: the monkey observes the scene, evaluates all the present cues and makes prediction about the compatible action that the observed agent will perform. In our model this corresponds to the transmission of activity from PFC to the first elements of the IPL chain that codes the hypothesized goal. Note that this input is sub-threshold and alone will not produce the activation of the target neurons. The presence of the cues and the simultaneous observation of the action will produce the activation above-threshold and transmission of the activity along the chains.

During the observation of a motor sequence executed by another individual, information about hand configuration and motion, very likely originating from STS (superior temporal sulcus) region [34], reaches specific pools in the chains. Elements composing the chains are activated step by step in relation to the execution of each motor act by the observed agent. It is possible that the observed agent changes his action goal in the course of the action (e.g. he places the food into the container rather than bringing it into the mouth). In this case there is a mismatch between the predicted motor act and the actual observed act. This mismatch will produce an interruption in the chain activity propagation due to the lack of the appropriate visual feedback signal. Thus, the corresponding pool in the chain will remain silent. This mechanism eliminates, during the action, the chains that do not represent the matching motor sequence and thus the wrong hypotheses are discarded.

When the observed agent terminates the action that is in accordance with the hypothesized intention, the matching chain will produce an output signal that is used by the corresponding Intention pool as a feedback concerning the correctness of the proposed hypotheses. This allows to update the internal association rules. In the visual task the uncertainty is much higher than in the motor task because the monkey has no direct access to the observed agent's intentions and decisions. Similarly to the motor task, if the cues are ambiguous or even contradictory (for example simultaneous presence of the food and of the object increases the degree of uncertainty), the range of possible predictions is wider and a greater number of chains will be activated in parallel.

### Learning new action sequences

The model presented in the previous sections assumes that action sequences are encoded as neuronal chains. An important issue is how these chains can be formed in an adult organism through a biologically plausible learning process and integrated into an already existing network. In this section we propose a simple learning mechanism that exploits a Hebbian mechanism in order to produce the linking of neuronal pools leading towards the construction of goal-directed neuronal chains.

Before explaining the learning mechanism it is important to consider that learning new actions involves several levels of the motor system: a more peripheral level concerning the correct muscle synergies, a more central level for the correct composition of motor acts, and a level of association between cues and actions.

In this section we will concentrate on the second level, i.e. the concatenation of motor acts, and will describe a learning mechanism based on the “hardwiring” of successful sequences.

We assume the existence of a brain area dedicated to the generation of new motor plans. Based on its known functional properties this area most likely corresponds to the prefrontal cortex [20], [35]. According to the proposed model, PFC is responsible for the evaluation of context, motivation and past events and can generate motor plans aimed at achieving specific action goals. According to this view, the sedimentation of motor sequences (within the IPL) is based, at the beginning of the generation of new action sequences, on the re-arrangement of the already present motor acts that must rely, at least in part, on a “trial and error” strategy. Motor acts representations are thus activated with a varying degree of randomness by the PFC and the outcome of the executed sequences is used to train the network. Successful sequences, i.e. those that lead to a goal and a reward (for example a piece of food), produce a bias in the decision mechanisms in PFC and are then repeated more frequently, thus being strengthened by means of a Hebbian-like learning rule. More precisely, synaptic connections between neurons of pools that are activated sequentially and that fire shortly after each other are strengthened due to spike timing dependent plasticity (STPD [36], see Materials and Methods for more details). Simulations show that this mechanism is able to produce chains in which the activation of one pool is possible only if the previous pool and a combination of sensory and proprioceptive signals are active. In other words, the propagation of activity through a chain is possible only if at least two information sources (e.g. preceding motor act and contextual cues) provide simultaneous input, as one source alone leads only to a subthreshold activation of the population.

### Simulation 3

In order to test the learning capabilities of our system we utilized a neural network with four neuronal pools, each one encoding a specific motor act, connected in an all-to-all fashion. Inter-pool weights are initially set to low random values (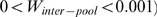). We trained the network simulating the PFC-driven execution of the (usual) “reaching, shaping, grasping, bringing-to-the-mouth” action sequence by providing the single pools with filtered activity profiles of the corresponding neurons recorded in vivo in the monkey IPL (see figure 4). We applied a Gaussian filter with 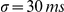, in addition to a baseline subtraction and a peak normalization to 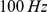. We recall that the duration of each burst is around 300 ms, and subsequent bursts overlap for approximately 80 ms.

**Figure 10:**
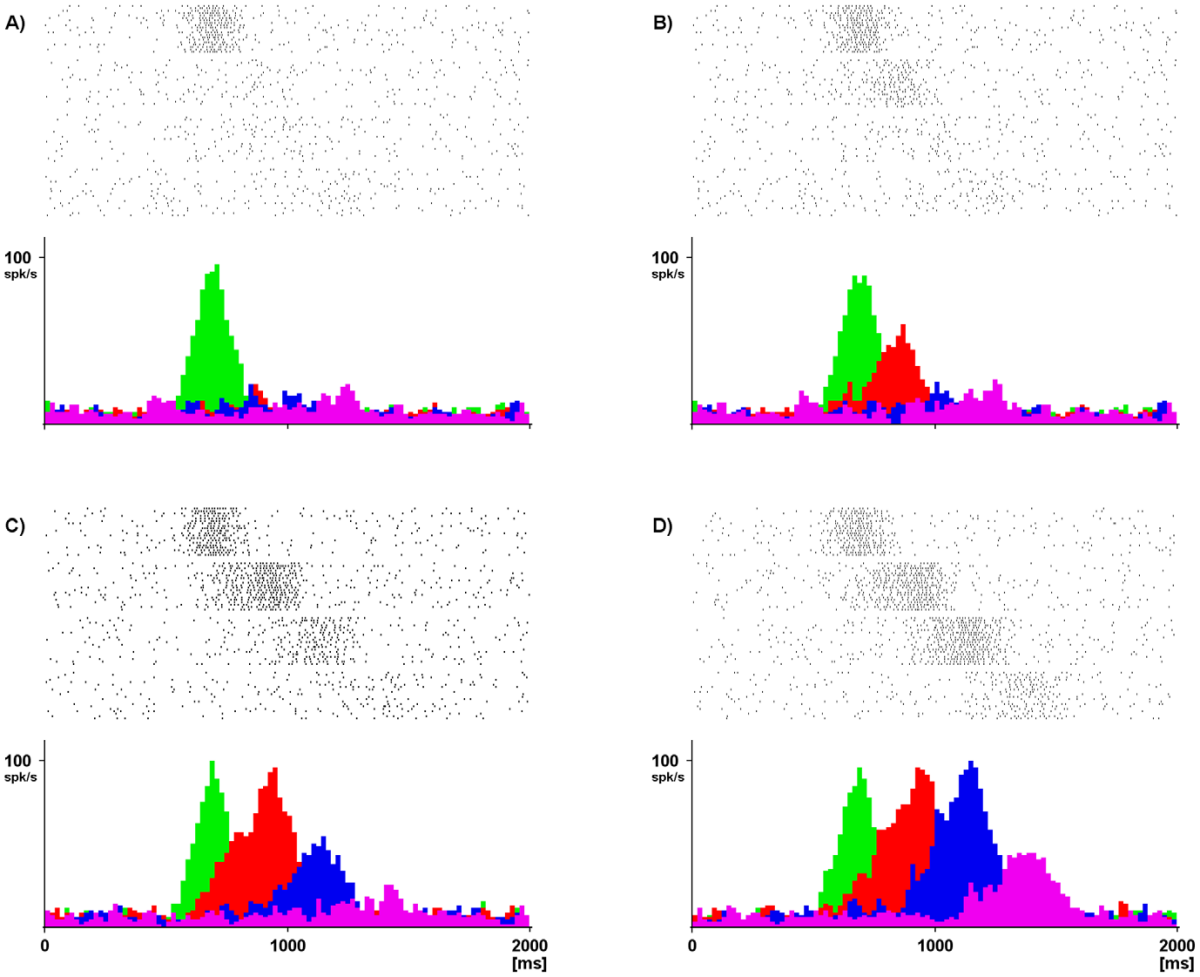
Representation of the responses of 4 neurons of the network at different moments of the learning phase. Panel A) response in the initial configuration; B) after 150 sessions; C) after 250 sessions; D) after 500 sessions. Color code as in Figure 4.

The strengthening of the synaptic connections takes places during the period of time in which the activity of one pool overlaps with the activity of the following one according to eq. 8 (see Materials and Methods). Figure 10 shows the various phases of the learning process of the network. The colored peaks represent the responses of four neurons coding subsequent motor acts as a reaction to the stimulation of the first pool of the chain (green). As learning proceeds, the connections between subsequent motor acts become stronger and pools tend to respond more.

## Discussion

The model proposed in this study shows that a network of spiking neurons organized in chains dedicated to specific goal-related actions is able to reproduce the behavior of motor neurons of the parietal cortex during action execution. Furthermore, this network is also able to reproduce the behavior of mirror neurons during both action execution and action observation.

In the present study we opted for a chain-organized structure instead of other types of configurations because in our view this better reflects the anatomo-functional organization of IPL, in which motor acts performed with different effectors such as the arm, the hand and the mouth are represented in a caudo-rostral sequence [5]. This anatomo-functional organization, in which adjacent and partially segregated motor fields are reciprocally connected, appears to be suitable for a sequential activation of motor acts in order to build goal-directed actions, such as reaching, grasping, bringing to the mouth and eating.

Previous studies have demonstrated that chained organizations [37], [38], distributed representations [39]–[43] and coupled oscillators [44] can be used for generating sequences. The synfire chain, first theorized by Abeles [37], [38] and then used in several studies [45]–[47], is the canonical topology for sequence generation. Theoretical and empirical studies have shown that synfire chains are robust for spike sequence generation [46], [48]. They have been also proposed to be the neural mechanism underlying, for example, the precise spike sequences observed in the zebra finch premotor neurons [49], [50]. The validity of synfire chains, however, is still an active topic of debate.

The model proposed in this work shares similarities with synfire chains in that a sequential activation of subpopulations of neurons occurs. However, differently from synfire chains, the neuronal activations occurring in our model do not require strict synchronicity, therefore the same sequence can be run with varying durations of the single motor acts. This happens because the propagation of activity is strongly regulated by external signals coming from motor and sensory areas. This feature makes our model also substantially different from simple pattern generators, such as those produced by coupled oscillators models for simulating lamprey swimming [44] and the aquatic and terrestrial locomotion of the salamander [51].

Another significant feature of the present model is its biological plausibility. This derives from several factors. First of all, the role and the connectivity of each network module is based on known functional and anatomical properties of IPL and of the areas linked with it. Among the latter there are areas providing visual signals on objects or biological stimuli, such as the inferior temporal cortex and the STS region, other providing somatosensory signals such as the superior parietal cortex and the secondary somatosensory area (SII), other sending motor signals, such as the PMv. Second, the integration of external sensory inputs and motor signals in the chains is based on the known sensorimotor integration mechanisms occurring in the IPL [3], [5]. Third, the temporal discharge profiles of the simulated neurons closely resemble those of the neurons recorded in IPL. Fourth, our model is conceptually grounded on the neurophysiological findings that the motor system is active not only during action execution, but also during action observation, leading to the proposal that action understanding derives from a mechanism matching others' actions on the observer's motor repertoire. Because of this, in the present model we have employed the same chain structure both during execution and observation of motor actions. Its working modality is determined by the different contributions coming from external sensory and motor areas.

Lastly, our model suggests a possible prefrontal selection mechanism at neuronal level, which can be involved also when multiple intentions or potential actions might occur simultaneously (e.g. blind task).

An important finding of the present study is that chains of neurons can be built through fast learning by means of a local reorganization of the network. This reorganization does not imply the generation of new chains but the strengthening of already existing connections and the weakening of others through Hebbian learning. This mechanism can explain the acquisition of new action sequences in adults as well as in children, using a basic repertoire of motor acts. Important factors that we did not consider in the present study probably influence this process, such as motivation, skills in the fine movement control, extension of the basic motor repertoire (for example by including tool use), memory span and capacity of flexibly assembling motor acts.

According to our model, the chain structured organization of IPL appears to be suitable for three main functions:

- Generation of context-dependent motor sequences. The link between contextual cues and motor acts and between these latter and the final action goal establishes associations between all of them. Once these links have been established such motor organization would facilitate the smooth execution of sequences of motor acts for the achievement of specific action goals.
- Motor imagery. During motor simulation, neuronal chains can run also without any overt motor output. This is possible by supposing that there exists an inhibitory mechanism that blocks the transmission of neuronal activity or decreases it below threshold levels before it reaches primary motor cortex or exits from it. Brain imaging studies show that PMC is active during motor imagery of intentional actions. Furthermore, recent neurophysiological data in monkeys demonstrate the existence in PMC of inhibitory mechanisms that are working during action observation [33]. We suggest that similar pathways are also active during motor imagery.

Since proprioceptive and other sensory signals are absent during motor imagery, the chains are “disconnected” from the external physical world. As proposed by the model, they are activated by the input coming from PFC and the missing sensory input is partially substituted by a signal provided by internal sensory copies associated to the corresponding action. A prediction of this model is that during motor imagery the propagation of activity can take place at a higher speed than during the motor or visual tasks because the real world time constraints are lacking and therefore the propagation depends only on internal dynamics.

- Action and intention understanding. Because of the dual property of mirror neurons, the same neuronal chain that is active during the execution of an action is also recruited when a similar action, performed by another individual, is observed. Since each chain corresponds to a specific goal, the state of the activity of neurons coding the first motor acts of the action sequence constitute an indication of the predictions of the observer (the monkey) concerning the intentions of the acting individual. On the other hand, when contextual cues are ambiguous or not present (e.g. the observer cannot see whether the performing individual is grasping food or an object) the model predicts an activation of the chains compatible with the two goals (eating and placing). Thus, in this case, the analysis of neuronal activity will not reveal to us any unambiguous prediction of the observer. There are situations in which only the initial part of an action can be observed and its outcome is hidden [52]. The model suggests that the missing part of the action is internally simulated, possibly according to the mechanism described in (B). In this case the activity within the chains represents the prediction of the observer concerning the action outcome.

One of the advantages of the current model is its intrinsic capacity of building new chains through simple and fast mechanisms, not requiring a substantial reorganization of connection in the network, but involving a redistribution of the strength of connections between the motor acts already present in the network. This process can explain how new action sequences can be created during child development or even in adulthood once the basic motor representations have already been acquired. The increase in the number of action sequences may expand the capacity of motor planning by enhancing the combinatorial power of the motor system.

Besides its relevance for explaining normal behavior, the model of action chain organization proposed here may have important implications for our comprehension of the mechanisms involved in brain dysfunctions connected to higher order motor impairments and intention understanding. Recent physiological findings demonstrated that while typically-developed children show an early activation of mouth opening muscles during a grasping-to-eat task, thus revealing the presence of a chain organization, this activation is very much delayed in high functioning autistic children [53]. Furthermore, this mouth activation is completely lacking in autistic children, but not in typically-developed children, when they are simply observing the same action made by another individual. These data have been interpreted as a deficit in the organization of motor chains in autism, that are not properly activated during both action execution and action observation.

According to our model, a reduced or absent capacity of propagating information along the action chains would produce two types of impairments: 1) A reduction or lack of fluency between the motor acts embedded in a sequence, very likely resulting in a fragmented or erroneous sequence; interestingly, patients with lesions to parietal and frontal cortex have been shown to have deficits in sequencing behavior (for a review see [8]). Among them, patients with ideational apraxia are unable to perform series of acts involving the use of objects, thus failing to correctly execute an action sequence. 2) A difficulty in understanding others' intentions, because the link between motor acts and action goal (intention) is weakened.

In conclusion, the proposed action chain organization appears to be a biologically plausible model able to reproduce motor dynamics in planning and organizing sequences of motor acts and to predict others' behavior. Dysfunction of this motor organization clearly compromises such capabilities, thus producing deficits typical of some human neurodevelopmental disorder.

## Methods

### Neuron model

The neural network implementation we adopted includes channels activated by AMPA, NMDA, and GABA receptors, producing a highly realistic simulation of the spiking activity. Neurons are described by a leaky integrate-and-fire model (see for example [54]) and are characterized by a resting (leak) potential 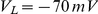, a firing threshold 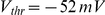, a reset potential 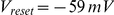, a membrane capacitance 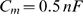, a membrane leak conductance 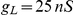, and a refractory period 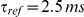. The corresponding membrane time constant is 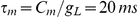 [55]. When the membrane potential 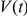 reaches the threshold 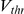 a spike is generated, and the membrane potential is reset to its default value 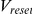.

**Figure 11:**
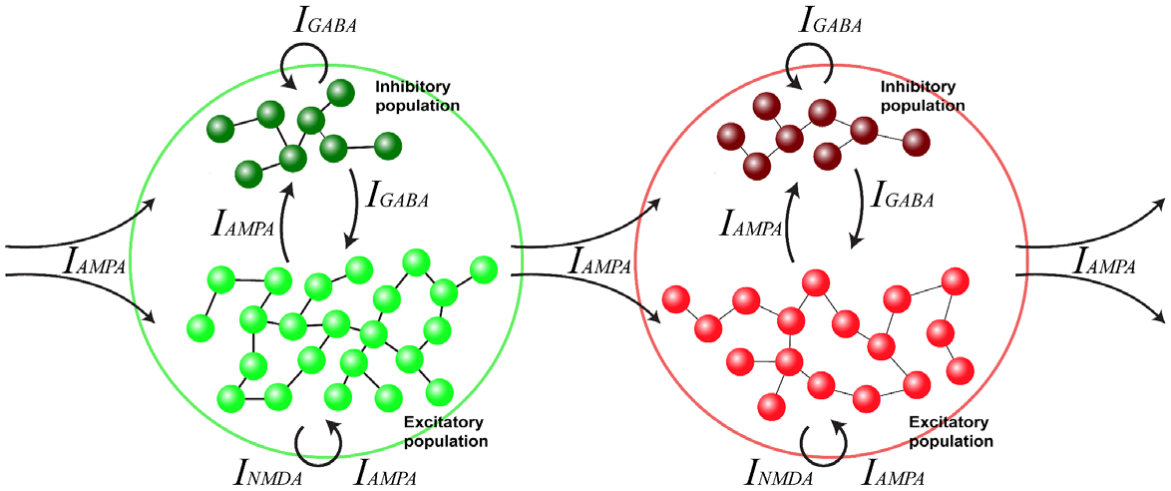
Schematic representation of two connected neuronal pools. Inhibitory neurons are represented as darker elements while excitatory neurons are represented as lighter elements. Arrows and the corresponding neurotransmitter indicate the type of connections between neurons within and outside the pool.

Below threshold, the membrane potential 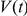 of a cell is described by:
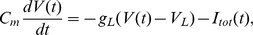
(2)where 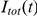 represents the total synaptic current flowing into the cell at time t. This current can be expressed as:
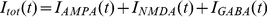
(3)i.e. the sum of glutamatergic excitatory components (NMDA and AMPA) and inhibitory components (GABA). We consider that external excitatory contributions are produced through AMPA receptors, while the excitatory recurrent synaptic currents are produced through AMPA and NMDA receptors (see Figure 11).

The current generated by each receptor of type X follows the general form [56]:
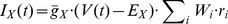
(4)where 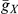 is the maximal conductance, 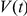 is the postsynaptic voltage, 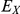 is the reversal potential, 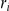 and 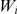 are the fraction of receptors in the open state and the connection strength with presynaptic neuron 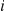 respectively. For AMPA and NMDA synapses 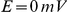 and for GABA synapses 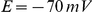. In case of NMDA an additional multiplicative term 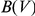 has to be added representing the magnesium block of the receptor channel. This block takes place extremely fast compared to the other kinetics of the receptor. The block can therefore be accurately modeled as an instantaneous function of voltage [57]:
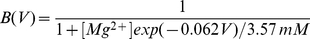
(5)where 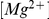 is the external magnesium concentration (in our case 1 mM).

The fraction 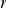 of the receptors in the open state is well described by the following first order kinetic equation:
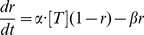
(6)The values of parameters 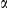, 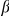 and 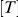 (neurotransmitter concentration) used in our simulations where taken from [56].

The total current 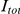 flowing into neuron 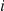 can also be written as the sum of different components:
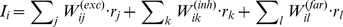
(7)where 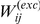 and 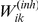 define the strength of local excitatory and inhibitory connections respectively, while 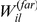 represents the connectivity to neurons belonging to other pools.

**Figure 12:**
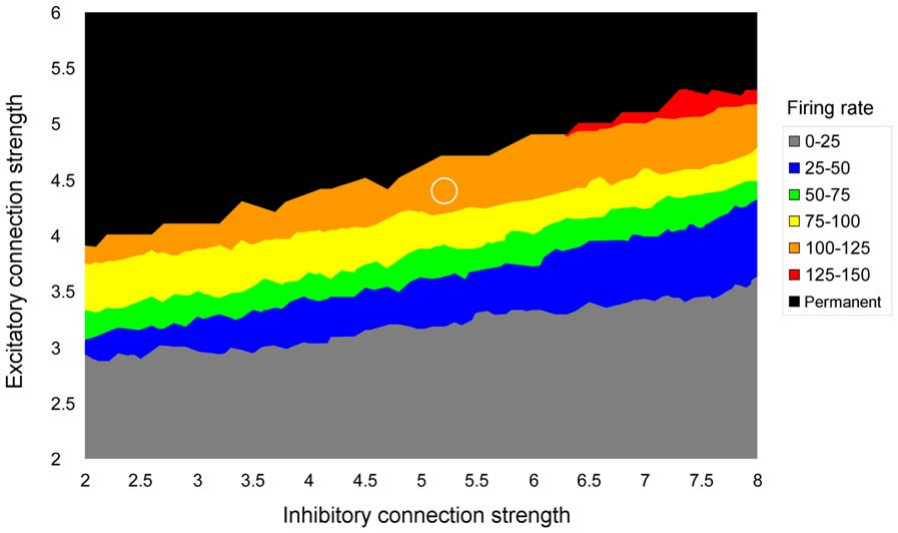
Representation of a pool's response as function of the excitatory and the inhibitory connections' strength. Different colors represent different levels of activity. The black area represents the combination of parameters that leads to a non-transient response of the pool. The white circle indicates the values of the connections' strength chosen in the simulations.

Examining the behavior of a neuronal pool as a function of the maximal excitatory 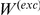 and inhibitory 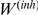 connectivity values (see Figure 12), the following values have been chosen in order to obtain neuronal pools that, when stimulated, produce a bell-shaped activity profile with a peak of approximately 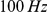: 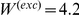, 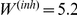.

### Spike timing dependent plasticity

The implemented learning rule is the spike-timing dependent plasticity (STDP) [36], [58]–[60], with dynamic synaptic plasticity. According to this rule the connection between two neurons is reinforced if the post-synaptic neuron fires shortly after the pre-synaptic neuron, while it is weakened when the pre-synaptic neuron fires after the post-synaptic neuron. An additional term has been added to this rule in order to take into account the dependency of the synaptic efficacy on the past activity the neuron [61], [62].

The general equation for the weight update can be written as:
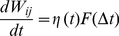
(8)where 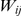 is the weight of the connection from neuron 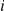 to neuron 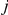, 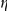 is the learning rate, 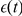 is the activity-dependent synaptic efficacy and 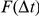 implements the spike timing dependent plasticity according to:
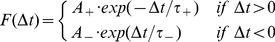
(9)with 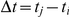, where 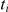 and 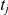 are the time of occurrence of the presynaptic and postsynaptic spikes, respectively. 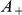 and 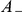 determine the maximum amount of synaptic modification (in our case 1), and the parameters 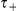 and 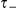 determine the ranges of pre-to-post synaptic interspike intervals over which synaptic change occurs (in our case 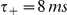 and 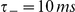).

Finally, for a given neuron the activity-dependent term has been implemented as:
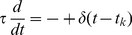
(10)where 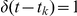 every time 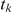 the neuron emits a spike, and 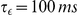 is the time constant of the process.

In order to avoid numerical instabilities associated with fast spiking activity, each neuron was simulated with a time step of 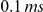 using the first-order Euler method.
